# Barriers and facilitators to breastfeeding during the immediate and one month postpartum periods, among Mexican women: a mixed methods approach

**DOI:** 10.1186/s13006-020-00327-3

**Published:** 2020-10-15

**Authors:** Sonia Hernández-Cordero, Ana Lilia Lozada-Tequeanes, Ana Cecilia Fernández-Gaxiola, Teresa Shamah-Levy, Matthias Sachse, Paula Veliz, Izchel Cosío-Barroso

**Affiliations:** 1 Universidad Iberoamericana, Ciudad de México, Mexico; 2 Ana Cecilia Fernández-Gaxiola- Universidad Iberoamericana, Ciudad de México, Mexico; 3 Centro de Investigación en Evaluación y Encuestas, Instituto Nacional de Salud Pública, Cuernavaca, Mexico; 4 Fondo de las Naciones Unidas para la Infancia, Ciudad de México, Mexico

**Keywords:** Breastfeeding, Bottle feeding, Health services research, Postpartum care, Social support, International code *of* Marketing of Breast-milk Substitutes

## Abstract

**Background:**

Evidence suggests that inadequate hospital practices, as well as sociocultural and community factors have detrimental effects on timely initiation as the first breastfeed within first hour after birth, and exclusive breastfeeding. The purpose of the study was to examine the factors that influence timely initiation of breastfeeding and exclusive breastfeeding at birth and 1 month postpartum in Mexican women delivering in public and private hospitals.

**Methods:**

Mixed methods were conducted between May and July 2017, including surveys (*n* = 543) and semi-structured interviews (*n* = 60) in the immediate (7 h) and intermediate (30 days) postpartum periods. Participants were women aged 15–49 years, in public and private hospitals, of urban and rural municipalities of Chihuahua and Puebla, Mexico.

**Results:**

Timely initiation was reported by 49.4% of mothers, and 34.7% reported that their children received infant formula at the hospital. Only 44.8% of women reported exclusive breastfeeding at 1 month postpartum. Timely initiation of breastfeeding was higher in women with vaginal delivery (62.1 vs 35.5%; *p* < 0.05) and those who received information during pregnancy (OR 1.07; *p* = 0.018). Exclusive breastfeeding at 1 month postpartum was related to older maternal age (OR 1.05; *p* < 0.001) and the fact that the mothers had received more information about breastfeeding during pregnancy (OR 1.13; *p* = 0.0001). Infant formula use was less associated with timely initiation (OR 0.46; *p* = 0.001). Participants in qualitative data identified the emotional, physical and economic benefits of breastfeeding, however, the perception about insufficient production of human milk, and the belief that infant formula is recommended, persists.

**Conclusions:**

Modification of hospital practices, such as decreasing the number of cesarean and the use of infant formula, as well as the support of the initiation and continuation of exclusive breastfeeding by health personnel and family members, could help increase breastfeeding practices in Mexican women.

## Background

The World Health Organization (WHO) recommends “breastfeeding within one hour of birth (timely initiation), and exclusive breastfeeding (EBF) during the first six months of an infant’s life with continued breastfeeding along with appropriate complementary feeding, to two years of age or beyond” [1]. Evidence suggests that inadequate hospital practices, and sociocultural and community factors have detrimental effects on timely initiation and EBF; these include, providing infant formula to new mothers, separating child and mother, not providing adequate information and support to mothers regarding breastfeeding, and sociocultural beliefs, among other effects [2, 3].

The WHO outlines a number of measures to contribute to the initiation, establishment, and continuation of breastfeeding, both within the health system and at the community level, in which multiple physical, psychosocial and emotional factors can influence the type of feeding the mother offers to the baby. The type of infant feeding offered is associated with “health inequalities, sociocultural issues, societal norms, and public policies administered through the provision of maternity care and support” [2]. In Mexico, mothers’ perceptions of low milk supply or low quality of human milk are common reasons for unsuccessful breastfeeding [4, 5]. Other reasons include maternal illness, mothers’ perception that their baby dislikes human milk [4], painful nipples and mothers’ return to paid work [5]. Social support also plays an important role in breastfeeding choices; specifically, support from family, friends, neighbors, and colleagues [5].

In 2015, in Mexico, 81.6% of deliveries occurred in public hospitals and timely initiation was especially low (32%) among mothers who were attended in the private sector, while in the public sector it was 54% [6]. A secondary analysis of the WHO’s Global Survey on Maternal and Perinatal Health showed that the prevalence of timely initiation was significantly lower among women with complications during pregnancy and caesarean delivery [7]. Likewise, cesarean births have been associated with delayed timely initiation and shorter breastfeeding compared to vaginal births, in Mexican women [8].

Mexican legislation states norms for healthcare during pregnancy, delivery, and the newborn period, that will allow them to successfully initiate and maintain EBF during the first 6 months of life, such as: adequate prenatal education to pregnant women about breastfeeding or rooming-in of mother-child dyad [9]. Furthermore, the 2014–2018 National Strategy for breastfeeding (Estrategia Nacional de Lactancia Materna) [10] focused on boosting the “Baby-Friendly Hospital Initiative” [1] and improving compliance with the “International Code of Marketing of Breast-milk Substitutes (BMS)” (the Code) [11] in the country. However, the regulatory framework to pressure the mass media and the infant formula industry was only recently established [12], and healthcare centers face difficulties to comply with BMS marketing ethics and regulations. A recent study provided evidence of violations to the Code in Mexico [13].

In Mexico, children are not receiving breastfeeding as currently recommended: particularly timely initiation, EBF, and duration of breastfeeding [4]. Therefore, we sought to examine the factors that influence timely initiation and EBF in the immediate postpartum period (up to 7 hours post-delivery) and at 1 month postpartum in Mexican women delivering in public and private hospitals of rural and urban communities.

## Methods

### Aim

To examine the factors that influence timely initiation and exclusive breastfeeding at the immediate postpartum period (up to 7 h post-delivery) and at one-month postpartum, in Mexican women delivering in public and private hospitals of rural and urban communities.

### Design

**Fig. 1:**
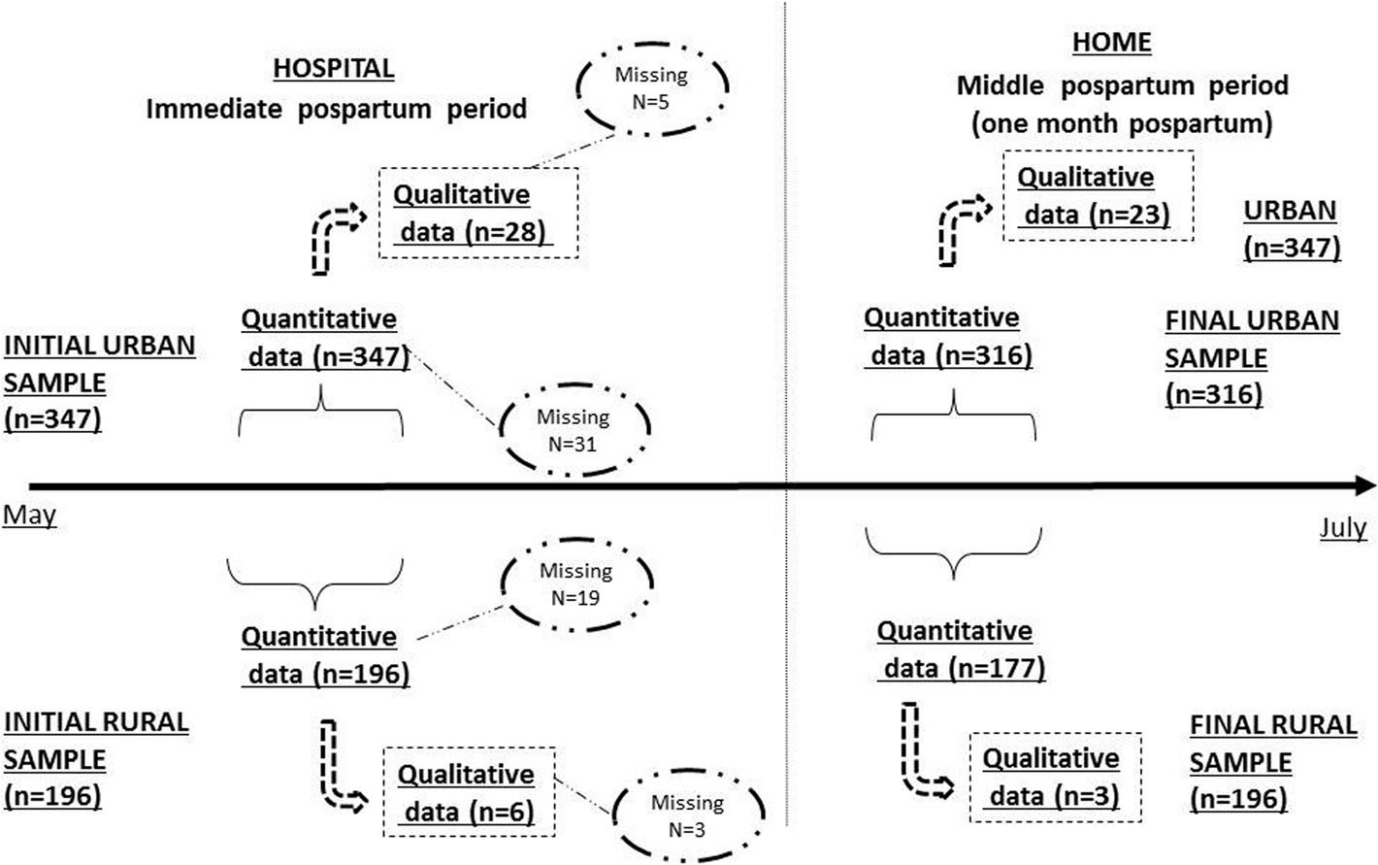
Timeline and mixed methods of the study, 2017. *In the immediate postpartum, 476 participants from the public sector and 67 from the private sector were recruited, with 434 from the public sector and 59 from the private sector remaining at the end of the study

Mixed-methods design, including surveys and semi-structured interviews with women who had recently given birth, in immediate (~ 7 h) and 1 month (30 days) postpartum. Both modes of data collection occurred at each time point (Fig. 1), and data were pooled and analyzed for each outcome of interest without comparison over time.

### Setting

Public and private hospitals from rural and urban communities in the States of Chihuahua and Puebla, Mexico. Detailed criteria for State selection, are explained elsewhere [13]. Briefly, states were selected as a follow-up of a 2016 study about violations of the Code [13]. Communities were selected randomly from those with the highest birth rates in each state (see Sample section); this increased the probability of finding participants in immediate postpartum period. The current study was conducted between May and July 2017. The protocol was approved by the Ethics Committee of the National Institute of Public Health.

### Sample

A sample size of 224 mothers per state was calculated, considering: a prevalence of 12% [4] of EBF at 1 month postpartum with a 95% confidence interval and a maximum estimation error of 6%; a design effect of 2.0 to correcting by cluster sampling (considered here hospitals as cluster) and 20% attrition rate. Public hospitals, with at least six deliveries per day, were selected randomly. A convenience sample was used for private hospitals, given the low rate of response, thus including only hospitals where Directors agreed to participate; for each case, a negotiation had to be made with the person in charge of authorizing us to conduct the study.

We identified potential participants in the delivery room and contacted them to invite them to participate within the first postpartum hours (Mean: 8.9 h, Standard Deviation: 7.3). Selection criteria included having a low-risk pregnancy, single pregnancy, gestational age ≥ 32 weeks, and maternal age between 15 and 40 years. Women were excluded if the mother or the newborn had a pathology that could limit breastfeeding, such as: HIV, Hepatitis B or C, Sepsis, Herpes, substances (drugs) use, among others.

All women received an explanation of the study procedures, and those who agreed to participate signed the consent form. Women took the survey and the interview (10% of the sample completed interview, this is 60 as whole across either both times throughout the study) at immediate postpartum in the recovery room, and in their home 1 month after delivery.

### Data collection

The survey comprised fixed-choice questions, including data on sociodemographics, health history, and breastfeeding topics (previous breastfeeding experience, reasons for breastfeeding, facilitators, having received information, benefits, duration and techniques for breastfeeding). For rural indigenous communities, health personnel translated interviews for indigenous-language speakers (*n* = 6). In addition, a checklist was adapted from a previous study [13] and used to identify the presence of visible or available BMS being advertised in the health unit. This was done by observation of different areas of the hospital.

Qualitative interviews were designed by expert personnel based on what has already been documented by previous studies and seeking to fill information gaps. The survey and interview guide were pilot tested in one hospital in Mexico City, during the training week. The field team consisted of six women with professional profiles in Nutrition, Public Health and Psychology. The study was conducted on hospitals’ regular days and operating hours for safety reasons for the field staff and excluded Sundays and evening hours.

The semi-structured interviews were used to explore practices, perceptions, and experiences regarding breastfeeding during the hospital stay, as well as beliefs and support received from family and community to initiate, continue and/or terminate breastfeeding, In addition, interviews were used to identify opportunities to improve breastfeeding practices at the immediate and 1 month postnatal periods; and, at 1 month postpartum, they served to explore maternal beliefs, as well as the support received from their families and community that facilitated or limited breastfeeding. The interview codebook used for the analysis of semi-structured interviews can be seen in Additional file 1.

Figure 1 describes the sample size at each time point of the study, by type of data collected and dropouts by rural and urban setting. In general, the main loss to follow-up was failure to contact the participant at home (wrong phone number, problems to communicate to the phone number provided during the first interview, or the participant was not found at home) or absence of the women at home when contacted, due to activities outside. It is worth mentioning that there was a higher dropout rate among women who participated in the survey plus the semi-structured interview, maybe because they invested more time.

### Measures

#### Quantitative data

Timely initiation was defined as the proportion of children who were put to the breast within 1 hour of birth. EBF was defined as the proportion of children who were fed exclusively with breast milk on the day prior to the interview at 1 month postpartum, without food and/or drinks consumed the previous day to the study or *status quo* method [14]. Infant formula use was defined as the proportion of children who were being fed with infant formula at the time of the study (1 month postpartum).

Information received during pregnancy was measured on a scale from 0 to 9, considering the number of questions used to collect these data. Thus, a point was recorded if women received advice on breastfeeding (e.g., health and nutritional benefits of breastfeeding, benefits of EBF, among others).

The following characteristics were treated as binary categorical variables: maternal age (0 = adolescent [< 19 years old], 1 = adult [> 19 years old]); indigenous (1 = Yes, 0 = No); educational level (1 = high school or less, 0 = beyond high school); marital status (1 = married/cohabitation with partner, 0 = single/separated or widowed); maternal employment (1 = Yes, 0 = No); type of delivery (1 = vaginal, 0 = cesarean); area of residence (1 = urban, 0 = rural); and type of clinic (1 = public, 0 = private). The Household Living Condition Index (HLCI) was calculated through principal components analysis [15]. The HLCI was based on housing conditions, possession of goods, and number of electrical appliances (the first component that accumulated 44.1% of the variability was chosen as an index). This index was considered a proxy of low, medium, and high socioeconomic status when divided into tertiles and was considered as categorical variable.

### Data analysis

#### Quantitative data

“STATA v 14.0” (StataCorp, College Station, TX, USA) was used for quantitative data. All the estimations of means and proportions considered the design of the sampling by conglomerates (at hospital level). The effect of the cluster sampling design (at hospital level) was considered. Descriptive statistics were used for population characteristics; chi-square, *t*-test, and confidence interval were used to report differences between groups (such as: age group or area) and the significance level was considered with *p* < 0.05. Logistic regression models were estimated to identify the odds ratio of timely initiation, EBF and use infant formula at 1 month postpartum. Factors associated with the outcomes of interest (timely initiation, EBF and use infant formula) were identified through stepwise command, which starts from a saturated model until it is reduced to a parsimonious model. The adjustment variables in the final models were mother’s age, indigenous ethnicity, educational level, marital status, employment, infant feeding practices and establishment of delivery, area of residence, and breastfeeding information during pregnancy.

#### Qualitative data

After the fieldwork, audios of interviews were transcribed according to specific guidelines that allowed for the standardization of information recording [16]. These guidelines were systematized and analyzed according to the principles of the Grounded Theory (GT) of Strauss and Corbin (2002) [17], particularly through content analysis based on the identification of properties and dimensions, based on the central themes of the interview and the data emerging in the interviews. An additional file shows the coding tree and code definitions in more detail (see Additional file 1).

## Results

### Quantitative results

Approximately 90% of the mothers interviewed at immediate postpartum participated in the 1 month follow-up (493 of 543). The average maternal age was 24.1 (± 6.17) years. Almost 57% of the women were housewives and most of them were Spanish speakers (93%). Fifty two percent had completed secondary school or a lower educational level. Most women (55.6%) had one child at the time of the study (Table 1).

**Table 1:** Sociodemographic characteristics and birth characteristics of the participants (*n* = 543)^a^

	Total (***n*** = 543)
**Mother’s age (years) (Mean ± SD)**	24.16 ± 6.17
**Gestational age (weeks) (Mean ± SD)**	38.8 ± 1.29
**Type of delivery (%)**	
Cesarean section	260 (47.88)
Vaginal	283 (52.12)
**Parity (Number of children) (%)**	
None	241 (44.38)
1	158 (29.10)
2 or more	144 (26.52)
**Indigenous**^b^ **(yes) (%)**	34 (6.27)
**Educational level (%)**	
Less than high school	303 (57.28)
High school or similar	162 (30.62)
Undergraduate or more	64 (12.10)
**Employment status**^c^ **(%)**	
Formal work	125 (23.06)
Informal work	41 (7.56)
Student	68 (12.55)
Housewife	308 (56.83)
**Type of healthcare institution (%)**	
Mexican Social Security	236 (43.46)
Ministry of Health	240 (44.20)
Private	67 (12.34)
**Area of residence**^d^ **(%)**	
Urban	347 (73.30)
Rural	196 (26.70)
**State (%)**	
Chihuahua	272 (50.09)
Puebla	271 (49.91)

^a^Proportion or mean ± SD are presented as indicated

^b^Indigenous, referring to women who speak an indigenous language

^c^Formal work = paid employment; Informal work = self-employment (merchant) or family business worker

^d^Urban = > 2500 inhabitants; Rural < 2500 inhabitants

#### Infant feeding practices

Infant feeding practices have shown to be different based on the mother’s age, the type and establishment of the birth, and the place of residence. In the sample, only 49.4% reported timely initiation, with rooming-in accommodation being a common practice (96%) (Table 2). The majority of the babies had been breastfed at least once (94.7%) prior to discharge from the hospital or 1 month after delivery (98.7%). One month after delivery, only 44.8% of the children received EBF and 47.7% were being fed with infant formula at 1 month postpartum.

**Table 2:** Infant feeding practices by maternal age, healthcare unit, type of birth and place of residence (*n =* 542)^a^

	Healthcare unit			Maternal age			Type of birth			Place of residence			Total
**Hospital data and infant feeding practices**	**Public** **(*n*** **= 476)**	**Private** **(*n*** **= 67)**	***P*** **value**	**Adolescents** **(*n*** **= 123)**	**Adults** **(*n*** **= 420)**	***P*** **value**	**Vaginal** **(*n*** **= 283)**	**Cesarean** **(*n*** **= 260)**	***P*** **value**	**Urban** **(*n*** **= 347)**	**Rural** **(*n*** **= 196)**	***P*** **value**	
**Birthweight (kg)** **(mean ± SD)**	3.17 ± 0.38	3.13 ± 0.32	0.43	3.10 ± 0.36	3.18 ± 0.38	0.04	3.14 ± 0.38	3.19 ± 0.37	0.12	3.17 ± 0.39	3.15 ± 0.39	0.69	3.16 ± 0.39
**Gestational age (38–40 wks)**	89.08	92.54	0.63	91.87	88.81	0.33	90.11	88.85	0.89	88.18	91.84	0.33	89.50
**Knowledge of breastfeeding support groups (yes)**	14.95	14.93	0.99	8.13	16.95	0.01	16.25	13.51	0.37	17.34	10.71	0.03	14.94
**Attended breastfeeding support group (yes)**	63.38	80.00	0.30	60.00	66.20	0.70	60.87	71.43	0.32	65.00	66.67	0.89	65.43
**Health personnel advising on breastfeeding**													
Doctor	42.62	50.00	0.23	63.64	39.29	0.31	50.00	35.48	0.48	47.62	36.00	0.06	43.28
Nurse	39.34	16.67		36.36	37.50		27.78	48.39		26.19	56.00		37.31
Health promoter or assistant	6.56	0		0	7.14		8.33	3.23		7.14	4.00		5.97
Other (nutritionist, social worker, midwife, psychologist or a combination of them)	9.84	16.67		0	12.50		11.11	9.68		16.67	0		10.45
Doesn’t know	1.64	16.67		0	3.57		2.78	3.23		2.38	4.00		2.99
**Timely initiation of breastfeeding** **(< 60 min)**	52.21	29.85	0.004	58.54	46.78	0.05	62.19	35.52	**0.0001**	41.91	62.76	**0.0001**	49.45
**Rooming-in (Yes)**	96.21	97.01	0.74	93.50	97.14	0.06	96.11	96.53	0.79	97.98	93.37	**0.006**	96.31
**Baby was given formula in the hospital (Yes)**	33.89	40.30	0.30	30.08	36.04	0.22	28.27	41.70	**0.001**	45.95	14.80	**0.0001**	34.69
**Mother had breastfed her baby at least once at the time of discharge from the hospital (Yes)**	96.08	84.75	**0.0001**	93.81	95.00	0.61	95.75	93.59	0.28	93.35	97.18	0.06	94.73
**Breastfeeding since mother left the hospital, to one-month postpartum (Yes)**	99.08	96.61	0.10	100.00	98.42	0.17	98.46	99.15	0.48	98.10	100.00	0.06	98.78
**EBF at one month postpartum**	48.03	21.05	**< 0.01**	24.78	50.93	**< 0.01**	46.48	43.10	0.45	36.98	58.76	**0.0001**	44.88
	**(*n*** **= 166)**	**(*n*** **= 36)**		**(*n*** **= 57)**	**(*n*** **= 145)**		**(*n*** **= 91)**	**(*n*** **= 109)**		**(*n*** **= 151)**	**(*n*** **= 51)**		**(*n*** **= 201)**
**Infant formula feeding at one month postpartum**	44.35	74.47	**0.0001**	66.67	42.99	**0.0001**	43.33	52.15	0.07	56.60	32.47	**0.0001**	47.73
**Information on breastfeeding during pregnancy (Scale: 0–10)**	6.18 ± 3.30	5.00 ± 3.39	**0.006**	5.02 ± 3.17	6.33 ± 3.32	**0.0001**	6.08 ± 3.24	5.98 ± 3.43	0.74	6.06 ± 3.26	5.99 ± 3.45	0.82	6.03 ± 3.33
**Techniques for breastfeeding initiation**	55.30	42.30	0.06	46.34	57.04	**0.03**	52.90	54.40	0.74	56.07	52.04	0.36	54.61
**Techniques for breastfeeding maintenance**	43.50	22.00	**0.002**	25.20	44.87	**0.0001**	42.40	39.30	0.47	40.46	40.31	0.97	40.41
**Support to maintain breastfeeding at one month postpartum**													
Health providers	17.92	21.74	0.66	16.67	18.92	0.05	16.13	20.39	0.27	9.80	21.38	0.38	18.37
Partner or spouse	11.56	4.35		0	14.19		10.75	10.68		13.73	9.66		10.71
Mothers /mothers in law of participants	48.55	43.48		58.33	44.59		54.84	41.75		50.98	46.90		47.96
Family/friends	20.81	30.43		25.00	20.95		18.28	25.24		23.53	21.38		21.94
Other^b^	1.16	0		0	1.35		0	1.94		1.96	0.69		1.02

^a^Proportions or means ± SD are presented as indicated

^b^Other, such as, support group on Facebook and a combination of health providers, family and friends

Abbreviations: *EBF* Exclusive breastfeeding

#### Received breastfeeding information and support

Approximately 14.9% of the women reported being aware of breastfeeding support groups at the hospital (Table 2). From those women, 65.4% reported receiving support from such groups. When examining other hospital practices, 34.6% mothers reported that their babies received infant formula during their stay at the hospital and 41.1% of the mothers reported this was due to similar aspects, such as: perception of milk being insufficient, infant preferences or cracked nipples. Crying baby (15.7%) and medical reason (14.3%) were reported as situations to use of infant formula in the immediate postpartum. Other reasons included need for mother rest and problems in breastfeeding technique (with 8.22% each one). Baby medical reasons and discontent with breastfeeding (altogether, 12.3%).

When analyzing the information received during pregnancy, there was an average of 6.03 (± 3.33) points on a scale of 0 to 9 (Table 2). The type of the information received was: importance of breastfeeding for the infant (85.1%), benefits of human milk (72.8%), and optimal duration of exclusive breastfeeding (~ 74%). There was less information about techniques to initiate (54.6%) and maintain breastfeeding (40.4%), including adequate suckling, position, or latching. Mothers or mothers-in-law of participants were the primary sources of support to continue breastfeeding (47.9%) (Table 2). A few mothers interviewed at the postpartum period reported that promotion of infant formula in the hospital was uncommon (< 2%). However, at 1 month postpartum, women reported receiving infant formula samples (4.26%) and gifts promoting infant formula (8.52%) at the hospital discharge. Women referred listening to or seeing infant formula publicity in their homes or communities (54.3%), being on television and radio the main sources (85.3%).

#### Factors related to initiation, continuation or cessation of breastfeeding

The logistic regression model indicated that babies born by cesarean delivery (Odds ratio [OR] = 0.37; *p* = 0.0001) were less likely to receive timely initiation. Attending a private hospital and living in a rural area were less likely to initiate breastfeed within the first hour after birth (Table 3). Women who delivered at a private hospital have 52% less chance of practicing EBF 1 month postpartum (OR = 0.48; *p* = 0.025), but those who had timely initiation (OR = 1.81; *p* = 0.005) and had information about breastfeeding during pregnancy (OR = 1.13; *p =* 0.0001) had greater chances of EBF 1 month postpartum. Multivariate analysis indicated that timely initiation protects against infant formula use (OR = 0.46; *p* = 0.001); in addition, it was negatively associated with the age of the mother (OR = 0.94; *p* = 0.001), and with receiving information about breastfeeding during pregnancy (OR = 0.88; *p* = 0.001). Moreover, living in a rural area was a protecting factor against the use of infant formula 1 month postpartum (Table 3).

**Table 3:** Logistic regression timely initiation (< 1 h), EBF and infant formula use at one-month postpartum^a^

	Timely initiation (< 1 h) (***n*** = 479)		EBF (***n*** = 484)		Infant formula use (***n*** = 411)	
	OR (95% CI)	***P*** value	OR (95% CI)	***P*** value	OR (95% CI)	***P*** value
Age group (adult women)^a^	**0.95 (0.92;0.98)**	**0.006**	**1.05 (1.02;1.09)**	**0.001**	**0.93 (0.90;0.97)**	**0.001**
Indigenous ethnicity (yes)	0.84 (0.56;1.25)	0.39	1.18 (0.79;1.77)	0.41	0.66 (0.42;1.04)	0.07
Educational level (Secondary or less)	1.12 (0.74;1.71)	0.56	0.91 (0.59;1.40)	0.67	0.87 (0.55;1.39)	0.58
Marital status (yes)	0.63 (0.33;1.18)	0.15	1.47 (0.77;2.84)	0.23	0.72 (0.35;1.47)	0.37
Employment (yes)	0.79 (0.52;1.21)	0.29	0.77 (0.50;1.19)	0.25	1.19 (0.74;1.89)	0.46
Timely initiation (< 1 h)	–	–	**1.81 (1.19;.2.75)**	**0.005**	**0.46 (0.29;0.73)**	**0.001**
Type of delivery (cesarean)	**0.37 (0.25;0.54)**	**0.0001**	1.18 (0.78;1.79)	0.41	1.06 (0.68;1.65)	0.78
Type of health unit (private)	**0.48 (0.24;0.94)**	**0.03**	**0.48 (0.23;1.00)**	**0.05**	1.95 (0.91;4.16)	0.08
Area of residence (urban)	**0.60 (0.39;0.93)**	**0.02**	**0.47 (0.30;0.72)**	**0.001**	**2.27** (1.40;3.66)	**0.001**
Breastfeeding information during pregnancy (Scale: 0 to 9)	**1.07 (1.01;1.14)**	**0.01**	**1.13 (1.06;1.20)**	**0.0001**	0.88 (0.82;0.94)	**0.001**

^a^Models adjusted for: mother’s age, indigenous, educational level, marital status, employment, infant feeding practices, type and establishment of delivery, area of residence and breastfeeding information during pregnancy; Comparison Group: Adolescents

### Qualitative results

During the study, 60 women were interviewed as whole across either both times: 29 interviews were conducted in rural areas and 22 in urban areas, nine of the latter being in private health units (Fig. 1).

Women’s ideas about the meaning and conceptualization of breastfeeding were explored (Table 4). The main attributes of breastfeeding included: mother’s perception of human milk as a primary food that enables the transmission of nutrients and the establishment of emotional bonds between mothers and infants. Results indicated that mothers do not differentiate between breastfeeding and EBF.

**Table 4:** Summary of results by categories emerging from qualitative interviews (*n* = 60), immediate and one month postpartum period^a^

Category	Quotations	Category	Quotations
Immediate Postpartum period		One month postpartum period	
**Meaning and concept of breastfeeding and EBF**	[the child can] grow up with breastmilk, so they grow healthy and strong, because it prevents diseases for him and me. (CHIMSSR03) … well, the mother transfers all the vitamins to the baby. (CHIMSSR08)	**Information from health personnel to maintain the breastfeeding at home**	Uh, no. They did not say anything to me. They did not even give me a diet. (PIMSSR87) … to nurse my baby as much as I could … they gave me positions like in the form of holding a ball, the common one, which is lying down and that’s it. (CHIMSSU06)
**Information and knowledge about breastfeeding in the hospital**	Well, that helps both me and my baby and what it is for … I have been told that right now, that since she (the baby) is little, the best thing is breastmilk. And it’s like a vaccine that helps her develop and I also have to give, well, I can breastfeed up to 6 months and from 6 months to 2 years (give her) breastmilk and a balanced diet. (PSSAR25) … they have told me not to eat irritating foods, … to keep a balanced diet, to drink plenty of water or plenty of fluids during the day … to drink something, even water before breastfeeding the baby. (CHSSAR35)	**Difficulties for breastfeeding at home**	Of course, I would like to fill him up myself. (CHIMSSU57) Mm, no, I have thought about giving her the powder, because I want to go out with my husband, because it is required to go out with your partner. I mean, I want to give her powdered milk to leave her with my sister. (CHIMSSR39) It was difficult because I had a lot of pain. (CHSSAU55) That they would teach me because I did not know anything at the beginning. (CHPR05)
**Indication of infant formula use**	Yes, the pediatrician of the evening [shift] told me to start with NAN1. (CHIMSSU57) Yesterday afternoon a doctor gave him formula because he (baby) was not filling up. (CHIMMSR11) A nurse, because last night that I breastfed her she did not get full and so they complemented. (CHIMSSU06)	**Facilitators for breastfeeding at home**	Well, I do not spend money and (the baby) is well fed. (CHIMSSR20) I do not have to prepare the portions or heat water. (CHIMSSR39) Well, I do not carry bottles and milk, and I do not have to get up at night to warm up milk. (CHIMSSU61) Right now, not because its easy for me, when she asks for it... well, the fact that I’m here, that I do not work and I have peace of mind and I do not find it annoying. (CHIMSSU61) Well, I almost always give him lying down, when I’m out in the street, I hold him inclined. [I do it because] well, they told me that children grow healthier and stronger and avoid diseases. (CHPR01) Sometimes my husband helps me to put the baby to the breast. (CHSSAU53)
**Reasons (“motivating factors”) for giving or not giving infant formula**	[Reasons for not giving formula] I have been told that bottles are bad, because they hurt their teeth and can give your baby colic. (PIMSSU66) [Reasons for giving formula] I think it was a decision of the pediatrician because they took him to the incubator and said that the most advisable thing was to give him formula. [the baby was] 3 h (in the incubator) because he lacked a little oxygen. (PPR86)	**Perception of recommendations/** **Opportunities to practice breastfeeding**	Good, it (breastfeeding) helped me put off some weight, because I gained almost the 19.8 pounds that are recommended. Well, I weighed 143.3 pounds and they tell me that I need to lose more (PIMSSR87). It’s cool because we can see the gestures the baby makes. (CHSSAR03) Well, do not despair when breastfeeding, because one still does not know how to place the baby. I learned alone to attach the baby, I put a cushion on my arm and shape my nipple. I make sure I am not holding him too tight so that he (the baby) does not suffocate. I make sure that he has the entire nipple in his mouth. Well, eating regularly, because I neglected myself a lot. (CHPR06) Well, give him breastmilk … to grow healthy, and milk is the first vaccine the baby receives. And when you are breastfeeding, you are cutting off the bleeding. (CHSSAR30)
**Facilitators to start breastfeeding in hospital stay**	… I have hospital gowns, they are comfortable, so there is not as much struggle to give him breast. (CHIMSSR08) Well, that the nurses and people (family) explain to me how to breastfeed my baby … well, [to have] a pillow and the bed. (CHSSAU35)		

^a^Quotes have been translated from Spanish to English as expressed by the participants

The main motivating factors for not using infant formula were the lack of interest in infant formula use, the belief that infant formula is harmful for infants’ teeth and the belief that infant formula causes colic. The primary reasons for using infant formula were the mothers’ need to adapt to breastfeeding and the mothers’ perception of insufficient milk supply. The main factors that contributed to timely initiation during their hospital stay included: nursing staff support, appropriate breastfeeding position, lack of financial resources to feed their babies, and access to drinking water to enhance milk supply.

The most frequent difficulties for breastfeeding 1 month after delivery were: 1) feeling of decreased milk supply; 2) the use of infant formula as an option when the mother is away; 3) sore and cracked nipples; and d) the need for home counseling support to understand the let-down reflex and how to achieve sufficient milk production. At home, mothers identified the following factors as facilitating EBF: 1) the belief that breastfeeding is easy; 2) low cost, practicality, and comfort; 3) a sense that their own nutrition and health helped breastfeeding practice; 4) a quiet and non-stressful environment (some stressful environments mentioned were public places and the workplace); 5) positions that facilitate breastfeeding; 6) the feeling of abundant milk production, and 7) support from their partners.

Similarly, the primary reason for infant formula use included: 1) feeling the infant was still hungry after breastfeeding; 2) continued use of a brand provided at the hospital; 3) it was perceived as beneficial by someone they trust (though not as good as human milk for avoiding diseases); and 4) the use of bottles with a pacifier-shaped nipple helps infants suck better. Relatives, friends, and health personnel were responsible for providing information on both breastfeeding and infant formula. Women also reported that they did not attend breastfeeding support groups, although they could identify some of them in their community.

Finally, women stated their own reasons for breastfeeding, including, easier weight loss and quicker recovery; being a positive, beautiful experience that creates bonds between mothers and their child; practicality; and that it made them happy. In addition, mothers acknowledged that breastfeeding: was their infants’ first vaccine is advantageous in disease prevention, and helped them grow strong and healthy. Mothers also acknowledged that: adequate latching is needed to avoid sore nipples; cushion use assists in holding up their arm during feeding; they must hug but not squeeze the infant; breastfeeding requires good body posture of the mother. Furthermore, mothers indicated that proper breastfeeding requires knowledge and patience.

Table 5 summarizes the barriers and facilitators for timely initiation at immediate and one-month postpartum and for EBF at 1 month postpartum, identified in this study.

**Table 5:** Summary of barriers and facilitators for timely initiation at immediate and one-month postpartum, and for exclusive breastfeeding (EBF) at one month postpartum (*n* = 60)

Facilitators	Barriers
***Immediate postpartum***	
a) Knowledge of breastfeeding emotional and physical benefits and techniques (information received during pregnancy) b) Breastfeeding counseling	a) BMS promotion b) Maternal and child hospital care practices (breastfeeding initiation after one hour after birth, infant formula feeding during first hour after birth, birth by cesarean section) c) Lack of support to women to initiate breastfeeding at hospital d) Lack of information about techniques to initiate and maintain breastfeeding
***One month postpartum***	
a) Knowledge of side effects of BMS b) Receiving information about breastfeeding during pregnancy c) Experiencing breastfeeding benefits d) Appreciation of breastfeeding practicality e) Appreciation of monetary savings with breastfeeding f) Family support (grandmothers, partner and other family members)	a) Milk insufficiency syndrome (perceived insufficient milk production) b) Belief that BMS complement breastfeeding c) BMS recommendation by health personnel and family members d) Sore/ cracked nipples e) Absence of breastfeeding counseling

## Discussion

This study examined the barriers and contributors of breastfeeding during the immediate and 1 month postnatal periods, in two states of Mexico. The rates of timely initiation (49.4%) and EBF at 1 month postpartum (44.8%) were similar to those reported at national level in the last UNICEF MICS (Multiple Indicator Cluster Survey– entitled in Mexico: “Encuesta Nacional de Niños, Niñas y Mujeres”), which were 51 and 38%, respectively [6].

We identified beliefs and practices that facilitate or hinder breastfeeding within the health system and in the community. Timely initiation and EBF facilitators were related to the information about breastfeeding or infant feeding practices that the mothers had received in critical times (such as pregnancy, early or 1 month postpartum), support either from health professionals or their family members, and the mothers’ previous experience with breastfeeding. The main barriers for timely initiation identified in this study were the maternal and child hospital care practices, including use of infant formula to feed newborns, BMS promotion within the hospital, lack of support to women to initiate breastfeeding, and births by cesarean section. In the case of EBF at 1 month postpartum, beliefs and perceptions about milk insufficiency and common difficulties faced during breastfeeding (such as sore/cracked nipples), as well the recommendation of BMS use by health personnel and family members, were identified as barriers.

Our findings are consistent with evidence from other countries, indicating that breastfeeding rates are low [18], and hospital practices that do not support breastfeeding are common, including: high rates of cesarean delivery [19], provision of infant formula, and lack of breastfeeding counseling [20–22].

In this study, timely initiation and EBF were lower in children born in private hospitals, among women who had undergone cesarean, and among those who lived in urban areas [7]. The negative association of cesarean with breastfeeding initiation has been described by others [7, 23]. A study in Nepal showed that mothers who delivered by cesarean section were less likely to initiate breastfeeding within the first hour after birth, whereas children that were not introduced to prelacteal feeds were more likely to receive timely initiation. The association of cesarean delivery and late initiation of breastfeeding (more than 1 hour after delivery) might be mediated by post-cesarean recovery period practices, which disrupt mother-infant interaction or inhibit infant suckling, both affecting lactation onset [8, 23]. There is scarce evidence about the differences between EBF and timely initiation in public versus private hospitals. However, one potential explanation of our findings is the higher rate of deliveries through cesarean section in private hospitals, as it has been documented in Mexico [24]. Another reason for a lower rate of timely initiation might be explained by the greater number of violations of the Code at private hospitals, such as: distribution of infant formula samples and advertising of BMS [13], which discourage all supportive practices towards timely initiation and exclusive breastfeeding.

Our results show a lower rate of timely initiation and EBF among adolescent mothers; this has been identified by other authors, showing maternal age as a significant predictor of duration of breastfeeding [25]. In a longitudinal cohort of pregnant adolescent females, in Connecticut, USA, 84% of adolescent mothers who initiated breastfeeding had stopped by 6 months postpartum, with an average breastfeeding duration of 5 weeks [26]. A potential explanation is that younger women usually report a lack of self-confidence for breastfeeding [5] and, as our results show, they receive less information about techniques for breastfeeding initiation and maintenance. These results are consistent with other studies of maternal and child healthcare among adolescents in Mexico, showing that adolescent mothers have less access to maternity care and, later, less healthcare access for their children [27].

One important timely initiation and EBF facilitator identified in our study was the information about breastfeeding or infant feeding practices that the mothers had received. The value of the knowledge regarding the benefits of and techniques for breastfeeding establishment for adequate infant feeding practices has been already documented. Patel, et al. [28] reported that counseling on breastfeeding during antenatal visits was associated with timely initiation of breastfeeding. For EBF, the results of a systematic review of Brazilian epidemiological studies by Boccolini et al. [29] show that there are different factors associated with exclusivity of breastfeeding, being maternal knowledge about infant health benefits of breastfeeding one of the EBF determinants, similar to what is shown by our results.

It is worth mentioning that even though in our study public hospitals had a higher rate of timely initiation and EBF, there are still several opportunities to improve hospital practices to enhance adequate breastfeeding practices. For instance, Mexican legislation states that routine use of infant formula should be avoided [9]. However, our results indicate the use of infant formula as a common practice, both in private and public hospitals, and the information women received during pregnancy and their hospital stay did not ameliorate the difficulties mothers experienced in breastfeeding and coping with the barriers to maintain this feeding practice. Moreover, the recommendation of the use of infant formula by healthcare professionals (physicians and nurses) was prevalent in our study as well. Women also mentioned that they used infant formula because they were having difficulties to establish breastfeeding or perceived their milk production as insufficient. On the other hand, only 18% of the mothers were advised to establish or maintain breastfeeding by health providers; this is contrary to what the evidence says about how improving breastfeeding knowledge and offering counseling on breastfeeding skills can improve breastfeeding practices, as we previously mentioned [30, 31]. In fact, results from a systematic literature review on the effect of breastfeeding interventions (timely initiation, exclusive, continued, and any kind of breastfeeding) provided by health systems indicated increased probability of any kind of breastfeeding by 47% (95% CI 1.29; 1.68); when counseling was provided in the home the effect was 17% more (95% CI 1.08; 1.27) [32].

The role of interpersonal relationships on breastfeeding practices, particularly the one concerning close family members (grandmother, mother-in-law, husband, female relatives) has been described by other authors [33, 34]. Consistent with what was reported in literature, women in our study reported that an important figure to maintain and support EBF at 1 month postpartum were family members such as mothers and mothers-in-law, and close family friends. One interesting finding was that some of the women interviewed mentioned their partner as an important source of support for breastfeeding. The role of the father on babies’ feeding practices might be related to mothers’ breastfeeding decisions and experiences, as it has been described by others [35].

Contrary to other studies [29], our results indicate that maternal educational level, marital status, socioeconomic level, and indigenous heritage were not related to timely initiation or to the duration of breastfeeding. It is possible that our study population is a homogeneous group [36].

Overall, there is a lack of support for the initiation and maintenance of breastfeeding during the immediate and 1 month postnatal periods. Such support should include theoretical and practical information; assurances; and space for women’s questions and concerns [31]. The findings of our study are consistent with other studies, indicating that breastfeeding women need support from healthcare providers, family members, and the community. Specifically, anxiety about the safety of the baby requires professional as well as family support, as mothers acquire new skills [25].

It is important to mention that the women admitted that they would recommend breastfeeding to others. Among the reported reasons for this were the psycho-emotional, physical, and economic benefits of breastfeeding, such as: affective mother-baby bonding, development of sense of protection, prevention of breast cancer, as well as saving time and money for the mother.

The study had some limitations, such as being an observational study and getting most of the sample from public hospitals. However, although there may be a selection bias due to the convenience sampling of private hospitals that authorized participation in the study, there were still differences between the public and private sectors: the latter, with the majority of hospital practices not facilitating or supporting EBF or timely initiation. Furthermore, the two states that were included in this study are located in the northern (Chihuahua) and south-central (Puebla) regions of the country, thus the results cannot be extrapolated. Our results are consistent with what has been found in other settings. However, it would be useful to have more studies identifying barriers and facilitators of adequate breastfeeding practices in other regions of the country, in order to have a better insight.

The strengths of the study, which are mainly that the mixed methods used give an extensive and detailed maternal perception of barriers and facilitators for timely initiation and EBF, are worth mentioning. In addition, the longitudinal approach is another important strength, as it gives the opportunity of having the perceptions of the same women on the different stages of the postpartum period (immediate and intermediate). Finally, we had a low dropout rate; it was lower than 10% in urban and rural areas.

## Conclusion

In conclusion, timely initiation and the duration of EBF may be improved by giving proper support to mothers from pregnancy, at early postpartum, and throughout the first months after delivery. In addition, findings are consistent with national and international protocols, suggesting that healthcare practices influence breastfeeding initiation, exclusiveness and duration. Finally, when implementing actions to increase timely initiation and EBF, special efforts should be targeted to women who: deliver in private hospitals, deliver via cesarean, are adolescent mothers or live in urban settings.

## Supplementary information

**Additional file 1.** Interview Codebook (primary properties and dimensions) used for the qualitative analysis of semi-structured interviews. This is an overview about interview codebook used in the qualitative analysis (coding tree and code definitions), which shows primary properties and dimensions of the categories of the qualitative data.

## Data Availability

The dataset or transcripts are available from the corresponding author upon reasonable request.

## Abbreviations

BMS: Breast milk substitutes
EBF: Exclusive breastfeeding
HLCI: Household Living Condition Index
MICS: Multiple Indicator Cluster Survey
OR: Odds ratio
The Code: International Code of Marketing of Breast-milk Substitutes
Timely initiation: Breastfeeding within 1 hour of birth
WHO: World Health Organization
