# Supplementary material for: Barriers and facilitators to breastfeeding during the immediate and one month postpartum periods, among Mexican women: a mixed methods approach
Source: Int Breastfeed J. 2020 Oct 15;15:87. doi: 10.1186/s13006-020-00327-3 (PMC7559131; doi:10.1186/s13006-020-00327-3)
Supplement: Supplementary file 1 — Additional file 1. Interview Codebook (primary properties and dimensions) used for the qualitative analysis of semi-structured interviews. This is an overview about interview codebook used in the qualitative analysis (coding tree and code definitions), which shows primary properties and dimensions of the categories of the qualitative data. [file 13006_2020_327_MOESM1_ESM.docx]

**Additional File 1. Interview Codebook (primary properties and dimensions) used for the qualitative analysis of semi-structured interviews.**

|  | | | |
| --- | --- | --- | --- |
|  | | | |
| **a. At 7 hours postpartum** | | **b. One month postpartum** | |
| **Categories** | **Description** | **Categories** | **Description** |
| Knowledge about | Set of knowledge, beliefs and experiences of women around the practice of breastfeeding. | Establishment of the breastfeeding | Mechanism for maintenance of exclusive breastfeeding (EBF) |
| Breastfeeding counseling at immediate pospartum | Information and knowledge provided by hospital health personnel regarding breastfeeding to support breastfeeding initiation during hospital stay | breastfeeding counseling | Information and knowledge provided by different members (i.e. health personnel, family members, relatives) regarding breastfeeding and infant feeding. |
| Hospital Practices | Actions and practices in the hospital implemented by health personnel in the immediate postpartum to promote timely breastfeeding initiation | Perception | Meanings/perceptions of breastfeeding among women who have had previous experience with this feeding practice. |
| Self-efficacy | Belief in one's own organizational capacity to manage possible situations and be successful in the breastfeeding. |  |  |

| **Interview Coding Guide (primary properties and dimensions)** | | | | | | |
| --- | --- | --- | --- | --- | --- | --- |
| **a.     At 7 hours postpartum** | | | | **b.    One month postpartum** | | |
| **Categories** | **Properties** | | **Dimensions** | **Categories** | **Properties** | **Dimensions** |
| Knowledge about breastfeeding | Concept or notion of breastfeeding | | --- | Establishment of the breastfeeding | Information provided by health personnel to continue Breastfeeding at home | Technique(s) |
|  | Physical and psychological benefits | | Mother |  |  | Counseling |
|  |  |  | Child |  |  | Person(s) Providing Information |
|  | Technique(s) | | Current. What it does or techniques that they doing about breastfeeding |  | Difficulties faced by the woman to continue breastfeeding | Ways to deal with it |
|  |  |  | To the future. What it considers it will do about techniques of breastfeeding |  | Facilitators to continue breastfeeding practice | --- |
|  | Reasons not to breastfeed | | --- |  | Reasons for discontinuation of the breastfeeding practice | Nipple cracking |
|  | Other feeding strategies | | --- |  |  | Mastitis |
| Breastfeeding counseling at immediate postpartum | Information received on breastfeeding | | Physical and psychological benefits (mother and child) |  |  | Infections or illness of the mother or baby |
|  |  |  | Suggested breastfeeding period |  |  | Recommendation from family members and/or health personnel |
|  |  |  | *EBF |  |  | Lack of time |
|  |  |  | Person(s) Providing Information |  |  | Other yonger children |
|  | Breastfeeding technical assistance from health personnel | | Who |  |  | Maternal work |
|  | Information received about other forms of feeding | | Who |  | Facilitators to continue breastfeeding practice after an interruption | --- |
| Hospital Practices | Promotion and use of infant formula (or breast milk substitute) | | Reasons. Causes or reasons that lead health personnel and women to promote and use formula | Counseling | Infant feeding practiced | EBF |
|  | Promotion and use of other prelacteal liquids m | | Causes and reasons to recommend used of other prelacteal liquids. |  |  | General breastfeeding |
|  | Mother-baby time together during hospitalization | | Motives. Causes or reasons that lead to keeping them together or not. |  |  | Introduction of complementary food |
|  | Advertising of formulas or substitutes | | Type of publicity |  |  | Providing infant formula (or breastmilk substitutes) |
|  | Influence of information and advertising on baby feeding practices | | By information received |  |  | Person(s) Providing Information |
|  |  |  | By publicity |  | Attend breastfeeding support group | Community |
|  | Facilitators for initiation of breastfeeding during hospitalization | | --- |  |  | Hospital |
| Self-efficacy | Intentionality to continue breastfeeding practice at home | | --- |  |  | Type of help or advice received given |
|  | Home breastfeeding Facilitators | | --- |  | Peer-to-peer Knowledge | Common or diverse breastfeeding facilitators |
|  |  |  |  | Perception | Recommendations of breastfeeding practices for women who have just had a baby | --- |
| **Emerging Codes** | | Ideas, thoughts or concepts with explanatory potential regarding the general or specific objectives of the study, but which were not necessarily considered. They may be of an innovative nature with respect to a hypothesis raised by the research objectives and questions. | | | | |
| Peer-to-peer counseling | | A set of popular knowledge that is transmitted from mother to first-time mother or not. | | | Experience about breastfeeding | Women's experiences during the first month pospartum. |
| Information for parents | | Popular knowledge that is transmitted between relatives (mother-mother, other women-mother). | | | Advertising of infant formulas | Degree of influence of advertising on breast-milk formulas and substitutes. |

***EBF=Exclusive Breastfeeding**
